# Non-invasive Determination of the Optimized Atrioventricular Delay in Patients with Implanted Biventricular Pacing Devices

**Published:** 2010-02-01

**Authors:** Thomas Deneke, Thomas Lawo, Stefan von Dryander, Peter Hubert Grewe, Alfried Germing, Eduard Gorr, Peter Hubben, Andreas Mugge, Dong-In Shin, Bernd Lemke

**Affiliations:** University Heart Center Bochum, University of Bochum, GER

**Keywords:** atrioventricular-delay, biventricular pacing, cardiac resynchronization, Doppler-echocardiography

## Abstract

**Background:**

Biventricular (BiV) is extensively used in the treatment of congestive heart failure but so far no recommendations for optimized programming of atrioventricular-delay (AVD) settings have been proposed. Can AVD optimization be performed using a simple formula based on non-invasive doppler-echocardiography?

**Methods:**

25 patients (ejection fraction 30±8%) received BiV ICDs. Doppler-echocardiographic evaluation of diastolic and systolic flow was performed for different AVDs (30ms to 150ms) and different stimulation sites (left ventricular (LV), right ventricular and BiV). The optimal atrioventricular delay was calculated applying a simple formula based on systolic and diastolic mechanical delays determined during doppler-echocardiography.

**Results:**

The mean optimal AVD was calculated to be 112±29ms (50 to 180ms) for BiV, 95±30ms (65 to 150ms) for LV and 75±28ms (40 to 125ms) for right ventricular pacing with wide interindividual variations. Compared to suboptimal AVDs diastolic optimization improved preejection and ejection intervals independent to pacing site. Optimization of the AVD significantly increased ejection time during BiV pacing (279ms versus 266ms; p<0.05). Compared to LV or right ventricular pacing BiV pacing produced the shortest mean pre-ejection and longest ejection intervals as parameters of improved systolic ventricular contractile synchrony. Diastolic filling times were longest during BiV pacing compared to LV or RV pacing.

**Conclusions:**

Individual programming of BiV pacing devices increases hemodynamic benefit when implementing the inter-individually widely varying electromechanical delays. Optimization applying a simple formula not only improves diastolic ventricular filling but also increases systolic functional parameters.

##  Introduction

Systolic resynchronization using biventricular (BiV) pacing has evolved as an established add-on therapeutic option in patients with symptomatic congestive heart failure. When pacing the left ventricle via the coronary sinus the contraction pattern of the interventricular septum and the contraction sequence of both ventricles is improved. This therapy has proven to increase systolic function (dP/dt) and cardiac function [1-5].

In patients with left ventricular pump failure conventional dual chamber pacemakers have been shown to alter hemodynamics in regard to the programmed atrioventricular delay (AVD). A long AVD leads to atrial contraction too early for optimal ventricular filling (loss of atrial kick). On the other hand a short AVD may lead to atrial contraction after closure of the atrioventricular valve (due to systolic increase of ventricular pressure) [6-10].

Different methods have been proposed to determine the most favorable (=optimal) AVD aiming at either optimizing systolic or diastolic cardiac function. Diastolic optimization of the AVD aims at restoring the atrial kick by coincidental timing of the end the left atrial systole and the mitral valve closure [2,6,11-17]. A simple formula incorporating time intervals measured during doppler-echocardiography has been evaluated for patients with IIIº AV block. When applying this formula the optimal AVD can be calculated from time intervals documented during long and short AVD pacing [18-23].

We studied the feasibility and efficacy of AVD optimization adapting a modification of a formula for optimal AVD programming in patients under biventricular stimulation with intrinsic atrioventricular conduction and left ventricular insufficiency. The effects on non-invasively determined parameters of cardiac systolic and diastolic function     were assessed. 

## Methods

25 consecutive patients were included after implantation of biventricular implantable defibrillators (ICD) (Medtronic InSync™ ICD) for chronic heart failure class II to IV in between 1999 and 2001. All patients gave informed consent; the protocol was evaluated by the institutional ethics review board.

**Figure 1:**
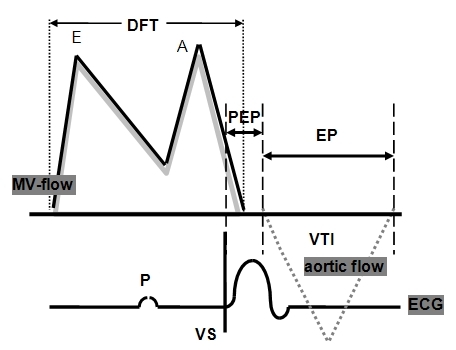
Schematic mitral valve and aortic doppler flow pattern and ECG: As measures of diastolic performance the diastolic filling times (DFT) were acquired and visual determination of E-wave (early filling) and A-wave (atrial contraction) was performed. As systolic functional parameter the preejection time (PEP) and the ejection time (EP) were assessed (VTI = aortic velocity time integral; P = P-wave, VS = ventricular pacing artifact).

In all patients a standard ECG (12-lead) and Doppler-echocardiographic studies were performed 30 days after implantation. Different AVDs were programmed (30ms, 80ms, 100ms and 150ms) for the 3 programmable stimulation sites (left ventricular = LV, right ventricular and biventricular = BiV). Fusion beats were excluded on the basis of QRS morphology. For each programming mitral- and tricuspid-valve and aortic valve Doppler-echocardiograms were performed using our Hewlett Packard Sonos 5500 echocardiography system with continuous display of ECG at a paper speed of 100mm/sec. Measurements were performed after a resting period of 1 minute in intrinsic rhythm. Prior to each measurement an adapting period of 1 minute was established. 3 consecutive atrioventricular and aortic valve flow profiles were analyzed and different time intervals were measured (see Figure 1) and the means were calculated.

### Diastolic measures (mitral valve pulsed-wave Doppler-echocardiography):

1. *Mitral valve diastolic filling time:* Measure of diastolic left ventricular filling.
2. *Tricuspid valve diastolic filling time:*Measure of diastolic right ventricular filling.

### Systolic measures (aortic valve continuous-wave Doppler-echocardiography):

1. *Pre-ejection time:*Indicating the intra- and interventricular conduction and contraction synchrony.
2. *Ejection time:*Measure of left ventricular ejection.

**Figure 2:**
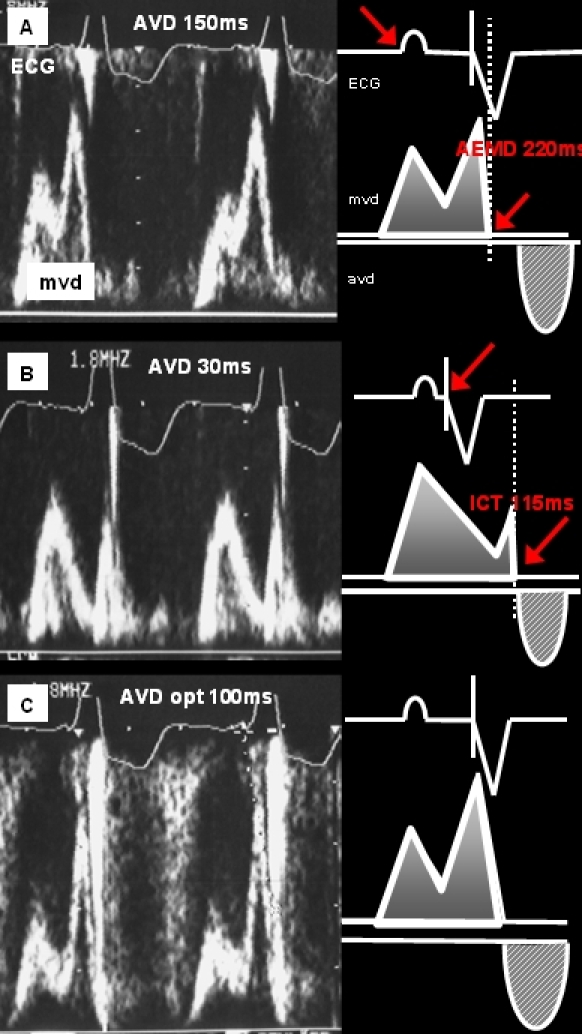
AVD optimization using non-invasive doppler-echocardiography during biventricular pacing: Mitral valve doppler (mvd)-echocardiographic findings (right) and schematic drawing (left). A. Long AVD pacing (AVD programmed at 150ms) and measurement of the atrial electromechanical delay (AEMD) of 220ms (= programmed AVD + interval between ventricular pacing artifact and mitral valve closure). B. Short AVD pacing (30ms) to determine the isovolumic contraction time (ICT) of 115ms. C. Calculation of the optimal AVD using the Ritter-Lemke formula (AVD opt = AEMD - ICT = 105ms). Programming the optimal AVD of 100ms leads to normalized mitral flow pattern and resynchronized timing of the left atrial and ventricular contraction (see text for details). (aovd = aortic valve Doppler, mvd = mitral valve Doppler).

As a next step the AVD producing the most favorable diastolic left ventricular inflow (= optimal) was calculated applying a simple formula (see Figure 2): Two steps of programming are needed to document the time intervals incorporated in the formula [18-22].

1. *Long AVD pacing (150 to 200ms) to determine the atrial electromechanical delay:*The atrial electromechanical delay constitutes the time interval in between the right atrial sensed electrical impulse to the end of the active mitral valve flow. This includes the interatrial conduction time and the electromechanical coupling of the left atrium. This interval is intraindividually fixed and has a wide interindividual variation.
2. *Short AVD pacing (30ms as the shortest programmable AVD) to determine the isovolumic contraction time:*The isovolumic contraction time is the time interval in between the ventricular electrical stimulation and the closure of the mitral valve due to the left ventricular systolic pressure increase. This interval can be measured when the atrial contraction is attenuated by the ventricular systole as documented in Doppler-echocardiography. This interval is intraindividually fixed and includes the conduction time from the pacing electrode to the left ventricle, the electromechanical coupling of the left ventricle and the duration from the beginning of the left ventricular systole to increasing intraventricular pressure above atrial pressure.
3. Calculation of the optimal AVD when subtracting the atrial electromechanical delay and the isovolumic contraction time (AVD optimal = Atrial Electromechanical Delay - Isovolumic Contraction Time).

When the calculated optimal AVD was different from the before programmed AVDs measurements were performed after optimized programming.

In each patient, measurements during optimized pacing (after programming the optimal AVD) were compared to the mean of the three AVDs (80ms, 100ms, 150ms) (= control) excluding the optimal AVD (if this was any of the ones testes: 80ms, 100ms or 150ms).

### Statistics

The means of the 3 consecutive measurements were calculated and the optimal AVD was calculated for each patients. Non-categorical variables of the different pacing site groups (LV, BiV, right ventricular) and different AVDs (30ms, 80ms, 100ms, 150ms and AVD opt) were compared using Student t-test. Treatment effects within the groups (optimized pacing versus control settings) were assessed by ANOVA analysis. A significant difference was proposed when p < 0.05.

## Results

25 patients with a mean ejection fraction pre-implant of 30% (± 8; range 19 - 41) and mean end diastolic diameter (echocardiography) of 66mm (± 7) were analyzed. Mean age was 65 (±10)years, 22 had left bundle branch block morphology whereas 3 had right bundle branch block morphology and mean intrinsic QRS width was 191 (± 38; range 155 - 264). Position of the LV pacing lead was posterolateral in 16 (64%), lateral in 6 (24%) and anterior in 3 (12%).

All patients were in sinus rhythm (mean heart rate 69±6bpm, range 58 - 86). Mean QRS width was significantly shortened during BiV pacing to 162ms (± 23) (p = 0.02), significantly longer during LV-pacing (238ms ±35) (p = 0.001) and significantly longer during right ventricular pacing (231ms ±39) (p = 0.006).

### Doppler-echocardiography and AVD optimization:

Heart rate did not significantly differ intraindividually (± 5 bpm) during any of the Doppler-echo studies. 

#### Diastolic parameters during Doppler-echocardiography

**Figure 3:**
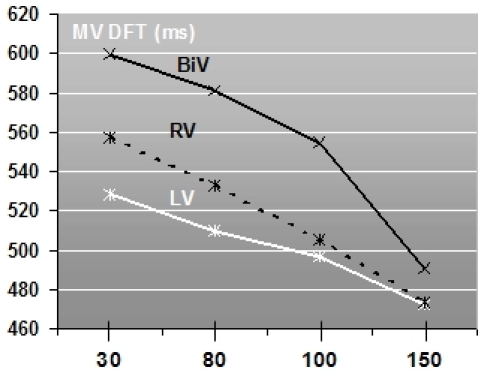
Mitral valve diastolic filling times in relation to the programmed atrioventricular delay and pacing site (BiV = biventricular, LV = left ventricular, RV = right ventricular): The mitral valve diastolic filling time shortens with consecutive AVD prolongation. BiV pacing produces the longest and LV pacing the shortest diastolic filling times (N = 19).

A consistent finding was the consecutive shortening of the diastolic filling times (over the mitral and tricuspid valve) when prolonging the programmed AVD (see Figure 3). LV pacing induced the shortest mean diastolic filling times at any programmed AVD compared to BiV and right ventricular pacing. BiV pacing produced the longest mean diastolic filling times irrespective of the programmed AVD. There was a wide interindividual range of measures of mitral and tricuspid valve diastolic filling time.

#### Systolic functional Doppler parameters

**Figure 4:**
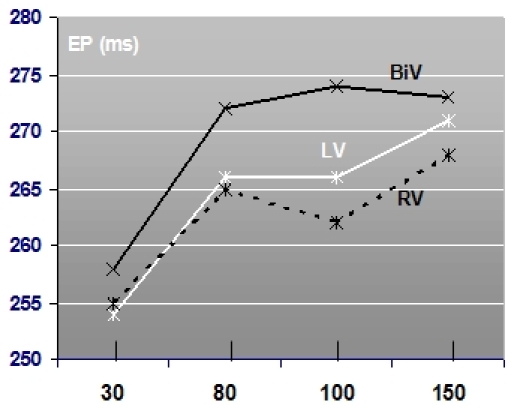
Mean ejection period in relation to AVD programming and pacing site: BiV pacing produces the longest ejection period correlating to improved systolic ejection independent to the programmed AVD (N = 19).

Longest mean ejection period resulted during BiV pacing compared to the monoventricular pacing modes independent to the programmed AVD. LV pacing seems to produce a longer ejection time compared to right ventricular pacing except at the shortest programmed AVD (30ms) (see Figure 4).

#### AVD optimization

1 of the 25 patients (4%) had no atrial contraction documented during transthoracic Doppler-echo. This patient was conclusively not eligible for optimization of the AVD. 24 of the 25 underwent Doppler-echocardiographic AVD optimization. The optimal AVD during BiV pacing was found to range in between 50ms to 180ms with a mean of 112ms (± 30). When correlating the optimal AVD to the intrinsic PQ-interval (optimal AVD-percentage) it was found to translate into 23% to 80%, indicating a shortening of the PQ-interval in between 20% to 77% to gain optimized BiV pacing. The optimal AVD during LV pacing was found to be 95ms (± 30) (p = 0.95 vs. BiV) (65ms to 150ms) and during right ventricular pacing was significantly shorter compared to BiV pacing at 75ms (± 28) (p = 0.003) (40ms to 125ms) (see Table 1).

BiV optimized pacing produced the most favorable systolic and diastolic functional Doppler parameter compared to LV and right ventricular pacing (see Table 1). Statistical significance was documented when comparing pre-ejection times during BiV and right ventricular pacing with a 13% increase in ejection time (159 ± 20 versus 182 ± 25, p = 0.009) (see Table 1).

#### Optimized pacing versus suboptimal AVD programming

**Figure 5:**
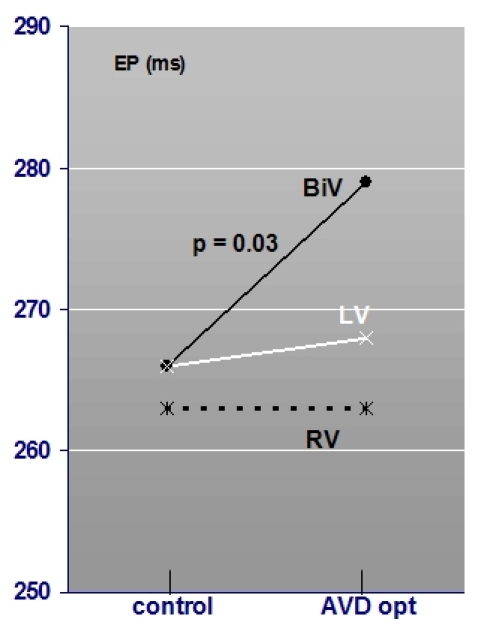
Mean ejection period during optimized pacing (AVD opt) compared compared to control settings during BiV pacing, LV pacing and right ventricular pacing (N = 18). Significant improvement in patients under BiV pacing.

AVD optimization compared to control settings during BiV pacing significantly increased ejection time from 266ms (± 30) to 279ms (± 25) (p = 0.03) correlating with an improved systolic ejection (see Figure 5). The pre-ejection time is shortened from 166ms (± 27) to 159ms (± 20) (p = 0.62). Diastolic filling times were found to be 568ms (± 137) under optimized pacing compared to 563ms (± 142) (p = 0.59) under suboptimal programming (tricuspid valve diastolic filling time: 601±137ms optimized versus 596±138ms; p =0.24).

During LV-pacing AVD optimization increased ejection time from 266ms (± 30) to 268ms (± 33) (p = 0.96) and pre-ejection time was shortened from 177ms (± 31) to 166ms (± 35) (p = 0.32) (see Figure 4). Diastolic filling times did not differ significantly.

During optimized right ventricular pacing ejection period stayed constant at 263ms (± 35) (263ms ± 29; p = 0.96) but pre-ejection time was shortened from 189ms (± 27) to 182ms (± 25) (p = 0.32) (see Figure 5). Diastolic filling times did not differ significantly in between settings.

## Discussion

There is still controversy about the effects of different programmings of the AVD in patients with pacing devices for chronic heart failure. Studies have emphasized the influence of the optimal pacing site in patients treated with biventricular pacing and there seems to be a beneficial effect of different AVD programmings modulating systolic ventricular function [2,16,23].

This study demonstrates improved systolic and diastolic function after non-invasive diastolic optimization on patients with implanted biventricular pacing devices. The optimal AVD was determined using a simple formula derived from findings in patients with complete heart block and is based on Doppler-echocardiographically measured electromechanical time intervals. Applying calculated optimal AVD programming diastolic filling is optimized and systolic function is improved. Improvements in systolic function may be due to a leftward shift in the Frank-Starling curve initiated by improved diastolic function. Although, optimizing the AVD may not lead to chronic increase in systolic function. Only a single report exists indicating functional improvement in biventricular pacing patients due to AVD optimization more than one month after optimization [24].

Different studies have evaluated the effects of different AVD optimization strategies. In most cases, the optimal AVD was determined by echocardiographic parameters of systolic function (e.g. left ventricular outflow). A recent study on 215 patients undergoing cardiac resynchronization therapy indicated the usefulness and safety of AVD optimization using Doppler mitral inflow data [25]. Controversy exists on the best strategy to identify the optimal AVD although tailoring optimal systolic Doppler-echo parameters appears to lead to better acute systolic performance. Our data suggest that optimizing diastolic left ventricular inflow will lead to acute improvements in systolic function [26,27]. In our study no comparison to other methods of AVD optimization was performed. We applied a simple equation for calculating the optimal settings for mitral valve inflow pattern.

We documented beneficial effects on systolic functional Doppler parameters indicating improved ventricular ejection and beneficial effects on intra- and interventricular electrical synchronization when coincidentally timing the end of the left atrial contraction and the beginning of the left ventricular systole. The optimal AVD can easily and non-invasively be calculated using a formula integrating two time intervals measured during long AVD and short AVD pacing. This formula was proposed by Ritter et al. in 1995 for patients with complete AV-block to resynchronize left atrial and ventricular systole enabling optimal ventricular filling and pre-load. However this formula has not yet been validated invasively [18-22]. In our study we transferred the initial formula to its electromechanical values and have documented the applicability in patients undergoing cardiac resynchronization therapy. Although, short intrinsic AV-conduction may involve a problem when long AVD pacing is performed during application of the formula optimization was effective in 96% of our patients. Only 1 patient was not eligible for diastolic AV-delay optimization because no left atrial contraction could be demonstrated.

Applying the calculated optimal AV-delay not only led to optimized timing of the atrial contraction just prior to the left ventricular systole but also produced changes in systolic functional Doppler parameters. During BiV pacing non-invasive diastolic AVD optimization significantly increased ejection time (5% increment compared to control settings). This implements significantly improved left ventricular systolic function only by modulating the AVD settings. Even though the correlation between ejection time and ejection volume is weak it is a measure of systolic ejection function indicating increased contractile function. Also AVD optimization led to shorter pre-ejection times documenting improved intra- and interventricular synchronicity. These findings are consistent with the documented changes of systolic function by Kindermann et al. when tailoring diastolic flow [3,21]. The optimal AVD was found to show wide interindividual variety independent to the stimulation site (for biventricular pacing in between 60 to 160ms). The differences are mainly due to the variations in electromechanical intervals incorporated in the Doppler-echocardiographic approach of optimizing diastolic flow. The atrial electromechanical delay is the time interval in between the right atrial sensed electrical stimulation and the end of the mitral valve flow due to left atrial contractile contribution. The longer the interatrial conduction time the longer the AV-delay optimum will be. In order to determine the atrial electromechanical delay the AVD should be programmed to the point where the mitral valve Doppler A-wave is not attenuated by the ventricular pressure rise. For determining the isovolumetric contraction time the shortest AV-delay possible is programmed and produces an attenuated A-wave (indicating the active closure of the mitral valve due to ventricular pressure rise) [18-22,28]. When considering these findings it becomes clear why individual programming of the AVD is superior to fixed AV-delay settings in this patient collective.

It seems possible to improve the beneficial effect of electric resynchronization therapy by careful and Doppler-echo guided individual programming of biventricular pacing devices. Even though, no guidelines for programming parameters for AVD optimization are available [16-14,16,24-28]. Our study demonstrates the feasibility of a simple method of diastolic resynchronization using Doppler-echocardiography in patients undergoing biventricular pacing.

It is well known that the effect of pacing therapies for chronic heart failure depend on selection of the pacing site. Whereas right ventricular pacing was shown to have controversial effects on left ventricular performance left ventricular based pacing seems to have beneficial effects on systolic function due to electrical resynchronization of the left ventricular contraction pattern [2,16,18-23]. In our study it is documented that BiV pacing compared to left ventricular only pacing favorably influences diastolic filling times. The longest diastolic filling was found during biventricular pacing irrespective to the programmed AVD and left ventricular pacing produces the shortest diastolic filling durations. This in contrast to the acute hemodynamic systolic benefit of left ventricular pacing. There appears to be a possible beneficial role of biventricular stimulation when considering diastolic hemodynamics [2,4,5].

As indicated in our study not only the filling volume but the preload which can be increased by optimal timing of the left atrial contraction and its contribution to ventricular filling is crucial for left ventricular performance. During optimized pacing the diastolic filling time is shorter than during short AV-delay pacing but correct timing of the left atrial contraction leads to optimized ventricular filling and readjusted mitral valve Doppler flow. These considerations explain the shorter diastolic filling times during optimized pacing compared to the suboptimal AVD programmings but still improved cardiac function during AVD optimization [11,12,16,18-22,28].

## Limitations

This study is limited by the small number of patients included making it impossible to determine any variables leading to changes in optimal AV-delay settings like LV-electrode position, cardiac disease or ejection.

As control the mean of AVD settings of 80ms, 100ms and 150ms were calculated and compared to optimized AVD pacing. This artificially constructed control setting is usually close to the range of the optimal AVD and therefore differences in between AVD settings may be marginal. On the other hand this control may resemble manufacturer's AVD settings implemented in the BiV pacing devices and therefore the analysis indicates the incremental benefit of AVD optimization compared to baseline settings.

We did not perform any invasive hemodynamic studies to validate the applied formula for AV-delay optimization. Even though the documented improvements in Doppler echocardiographic functional parameters indicate hemodynamic benefit this has not been clinically tested. In addition, no comparative study to other methods of AVD optimization was performed. It remains unclear, whether the proposed formula leads to the highest benefits or if other strategies (e.g. tailoring systolic Doppler-echo parameters) may further increase hemodynamics.

Especially in the latest pacing devices for biventricular resynchronization not only atrioventricular delay but also interventricular delays are programmable. Therefore there is a definitive need for guidelines or recommendations how to program different parameters of diastolic and systolic synchronization in these devices.

## Conclusions

Individual programming in patients under biventricular stimulation can increase the benefit of the resynchronization therapy when implementing the differences in electromechanical delays. Optimization of the AVD integrating these individual intervals can easily and non-invasively be performed using Doppler echocardiography applying a simple formula. Optimized pacing cannot only improve diastolic but also optimize systolic functional Doppler parameters.

## Figures and Tables

**Table 1:**
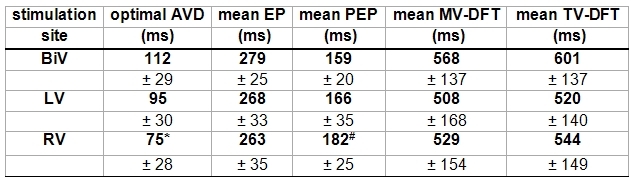
Mean doppler-echocardiographic parameters (± standard deviation) under optimized pacing at different stimulation sites of 24 patients

BiV = biventricular, LV = left ventricular, RV = right ventricular) (AVD = atrioventricular delay, EP = ejection period, PEP = pre-ejection period, MV-DFT = left ventricular diastolic filling time, TV-DFT = right ventricular diastolic filling time. * = p < 0.05 vs. BiV; # = p < 0.01 vs. BiV.
